# ﻿New insights into the chromosomes of stoneflies: I. Karyotype, C-banding and localization of ribosomal and telomeric DNA markers in *Skwalacompacta* (McLachlan, 1872) (Polyneoptera, Plecoptera, Perlodidae) from Siberia

**DOI:** 10.3897/compcytogen.18.115784

**Published:** 2024-01-25

**Authors:** Alexander Bugrov, Tatyana Karamysheva, Olesya Buleu

**Affiliations:** 1 Novosibirsk State University, Pirogova Str. 2, Novosibirsk 630090, Russia Novosibirsk State University Novosibirsk Russia; 2 Institute of Systematics and Ecology of Animals, Russian Academy of Sciences, Siberian Branch, Frunze str. 11, 630091, Novosibirsk, Russia Institute of Systematics and Ecology of Animals, Russian Academy of Sciences Novosibirsk Russia; 3 Institute of Cytology and Genetics, Russian Academy of Sciences, Siberian Branch, Pr. Lavrentjeva 10, 630090, Novosibirsk, Russia Institute of Cytology and Genetics, Russian Academy of Sciences Novosibirsk Russia

**Keywords:** 18S rDNA repeats, C-banding, FISH, Plecoptera, karyotypes, telomeric (TTAGG)_n_ DNA repeats

## Abstract

This study provides data on chromosome number (2n♂♀=26), sex determination mechanism (XY♂/XX♀), C-banding pattern, distribution of clusters of telomeric TTAGG repeats and 18S ribosomal DNA in the karyotype of the stonefly *Skwalacompacta* (McLachlan, 1872). For the first time in the history of stoneflies cytogenetics, we provide photos of the chromosomes of the Plecoptera insects. The karyotype of males and females of *S.compacta* consists of 12 pairs of autosomes. Three pairs of large autosomes and four pairs of medium-sized autosomes are subacrocentric. The remaining pairs of autosomes are small, with unclear morphology. Pericentromeric C-bands were revealed in all autosomes. The sex chromosomes are also subacrocentric. The short arms of X and Y chromosomes are entirely heterochromatic and are rich in ribosomal DNA sequences. In the X chromosome this arm is larger than in the Y chromosome. It is likely that this arm associated with the nucleolar organizer (NOR). Telomeric DNA (TTAGG)_n_ repeats were detected in the terminal regions of all chromosomes.

## ﻿Introduction

Plecoptera or stoneflies are amphibiotic insects distributed worldwide, except for Antarctica (Zwick 2000). Currently, about 3,700 species from 17 families of the stoneflies have been described (Fochetti and Tierno de Figueroa 2008; DeWalt et al. 2023).

To date, the stoneflies remain one of most poorly cytogenetically studied groups among the Polyneoptera. The karyotypes of only sixteen Plecoptera species from Europe, North America and Japan have been described (Nakahara 1919; Junker 1923; Itoh 1933; Matthey and Aubert 1947). These studies have resulted in information on karyotypes and sex determination mechanisms in this group of insects. For more than 70 (!) years, there has been no new information on the karyotypes of these insects. In reviews of sex chromosome evolution often refer to stoneflies as insects with highly diverse karyotypes and chromosomal sex determination systems (White 1973; Blackman 1995; Blackmon et al. 2017). Our research group has devoted several years to studying the evolution of sex chromosomes in grasshoppers (Bugrov and Grozeva 1998; Bugrov et al. 2001; Bugrov et al. 2016; Jetybayev et al. 2017; Buleu et al. 2020), and therefore we could not help but pay attention to the information about the intriguing variety of cytological mechanisms of sex determination in stoneflies. Taking into account the above, we set out to study the karyotypes of *Skwalacompacta* (McLachlan, 1872) using cytogenetic methods that have not previously been used in the practice of cytogenetic analysis of this group of insects.

The first paper in our planned series of studies is devoted to the description of the karyotype of the stonefly *S.compacta* from the Izdrevaya River in the vicinity of Novosibirsk.

To study the karyotype of *S.compacta*, we used the C-banding method to determine the localization and size of heterochromatic blocks in chromosomes and fluorescence *in situ* hybridization (FISH) with telomeric (TTAGG)_n_ and 18S rDNA probes to detect the localization of functionally important regions in autosomes and sex chromosomes. The choice of these molecular markers is determined by knowledge of their important functional role in the genome and information on the localization of telomeric DNA and ribosomal DNA in the chromosomes of many insect species (Frydrychová et al. 2004; Cabrero and Camacho 2008; Sharakhov 2015; Kuznetsova et al. 2019).

## ﻿Material and methods

### ﻿Material collection

Nymphs of the *S.compacta* of different ages were collected during the spring and autumnal season (2020–2022) in Izdrevaya river flowing within the city Novosibirsk (GPS coordinates 55.0018°S/N, 83.2156°W/E). The material for studying the karyotype of this species were testes and ovarioles of about 100 larvae.

### ﻿Methods

#### ﻿Chromosome preparations, C-banding and FISH

**Figure 1.**
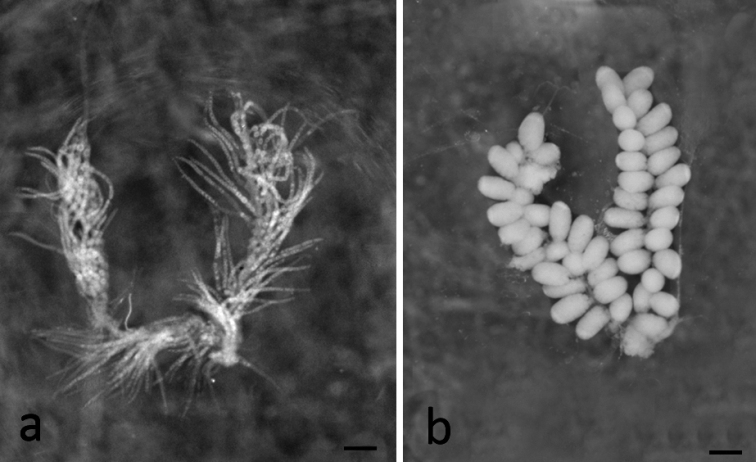
Ovaries (**a**) and testes (**b**) of larvae *Skwalacompacta*. Scale bar: 1 mm.

Prior to chromosome preparation, *S.compacta* larvae were stored in a refrigerator at 2–4 °C. Chromosome preparations were made from testes and ovaries of the larvae (Fig. 1). For this purpose, 0.1% colchicine solution was injected into the abdomens of *S.compacta* larvae. After 4–6 hours, the gonads were removed and placed in 0.9% sodium citrate solution for 15–20 minutes, and then fixed in freshly prepared ethanol : glacial acetic acid fixative (3:1) for 10–15 minutes. Fixed gonads were dissected using needles on pre-cleaned glass slides in a drop of 60% acetic acid. Finally, the cells were spread on the slide on heat plate at 65 °C.

C-banding of chromosome preparations was performed according to Sumner’s protocol (1972) with minor modifications. Slides were treated with 0.2 N HCL for 15–30 min, then rinsed with distilled water and dried at room temperature. Then slides were incubated in saturated Ba(OH)_2_ solution at 60 °C for 3–5 min, rinsed with water and placed into 2×SSC at 60 °C for 60 min. After washing in distilled water, slides were stained with 2% Giemsa solution in Sorensen’s phosphate buffer 30 to 60 min.

Fluorescence *in situ* hybridization (FISH) with telomeric (TTAGG)_n_ DNA and 18S rDNA probes was performed following the protocol of Pinkel et al. (1986) with modifications described in Rubtsov et al. (2000).

Telomeric repeats (TTAGG)_n_ were generated by non-template PCR with primers 5’-TAACCTAACCTAACCTAACC-3’ and 5’-TTAGGTTAGGTTAGGTTAGG-3’. Further labelling with Tamra-dUTP (Biosan, Novosibirsk, Russia) was performed in 33 additional PCR cycles as described previously (Sahara et al. 1999).

The rDNA probe was obtained as previously described by Jetybayev et al. (2017). Unlabelled ribosomal DNA probe was generated by polymerase chain reaction (PCR) according to Jetybayev et al. (2017). The fragments of the 18S rDNA were labelled in additional PCR cycles with Fluorescein-12-dUTP (Biosan, Novosibirsk, Russia) and mixed into a single ribosomal DNA probe.

Microscopic analysis was performed at the Centre for Microscopy of Biological Objects of SB RAS (Novosibirsk, Russia). Chromosomes were examined with an Axio-Imager M1 (Zeiss, Germany) fluorescence microscope equipped with filter sets #49,#46HE, #43HE and a ProgRes MF (MetaSystems GmbH, Germany) CCD camera. The ISIS5 software (METASystems GmbH, Germany) package was used for image capture and analysis.

## ﻿Results

**Figure 2.**
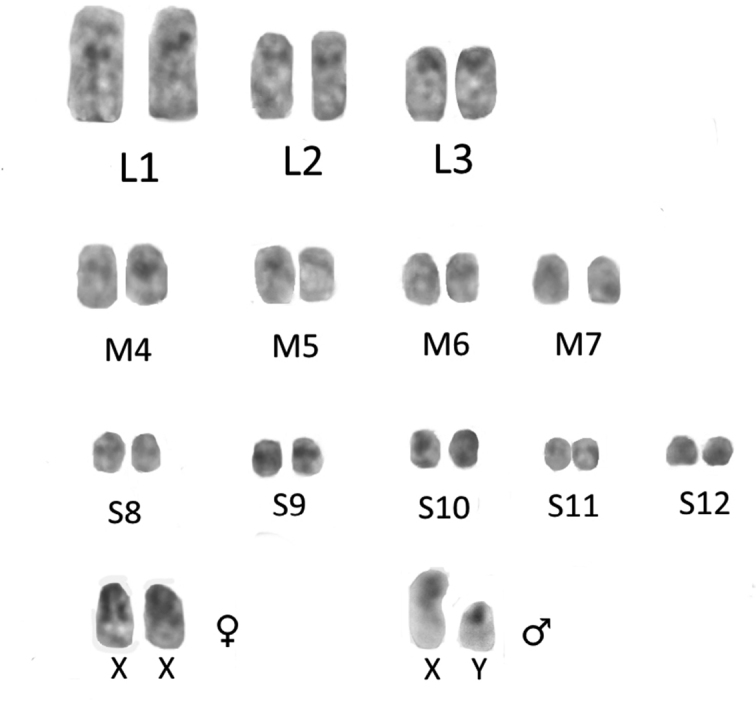
Joint karyogram of oogonial metaphase and spermatognial metaphase of *Skwalacompacta*. L – large, M – medium, S – small autosomes.

**Figure 3.**
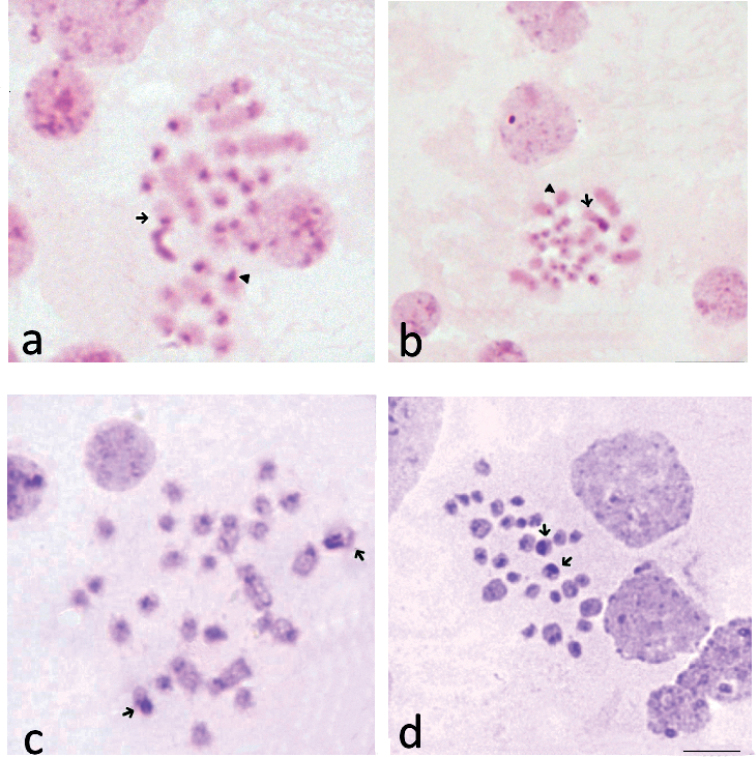
C-banded spermatogonial prometaphase (**a**), spermatogonial metaphase (**b**) and oogonial prometaphase (**c**), oogonial metaphases (**d**) of *Skwalacompacta*. Arrows – indicate X chromosomes. Arrowheads – indicate Y chromosomes. Scale bar: 5 μm.

The karyotype of males and females of *Skwalacompacta* consists of 12 pairs of autosomes. Three pairs of large autosomes (L1–L3) and four pairs of medium-sized autosomes (M4–M7) are subacrocentric. The remaining pairs of autosomes (S8–S12) are small, with unclear morphology. Pericentromeric C-bands were revealed in all autosomes (Figs 2, 3).

In the male karyotype, in addition to 12 pairs of autosomes, there are two heterosomes, which differ in morphology and size. The large heterosome is two-armed (Figs 2, 3a, b). One arm is entirely heterochromatic. The second arm is predominantly euchromatic, with a C-block localized in the proximal region. In spermatogonial prometaphase the size of heterochromatic arm can vary (Fig. 3a, b). The second heterosome is subacrocentric. According to the size and ratio of euchromatic and heterochromatic regions, one arm of this heterosome is morphologically homologous to the large arm of the large heterosome. The smaller arm of this heterosome is completely heterochromatic (Figs 2, 3a, b).

In the female karyotype, there are 13 pairs of chromosomes, one of which has a large heterochromatic arm in each homologue. Heterochromatic arms in these chromosomes can vary in size at different stages of oogonial metaphase, as is the case in the large male heterosome during spermatogonial metaphase (Fig. 3c, d).

A comparative analysis of the morphology and behavior of the heterochromatic regions of the large heterosome in males and the mentioned pair of chromosomes in females suggests that these are sex chromosomes. Based on this comparative analysis of the heterosomes, it can be concluded that the mechanism of chromosomal sex determination in *S.compacta* is XY in male and XX in female.

**Figure 4.**
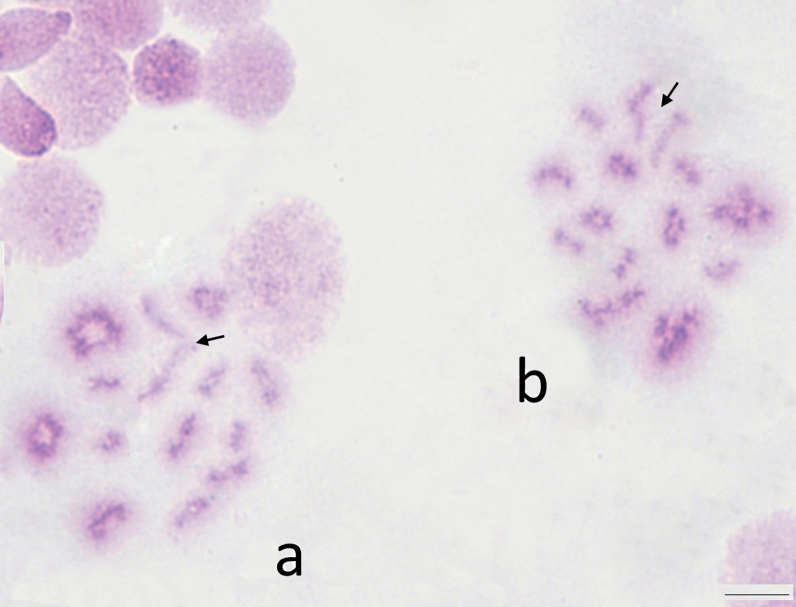
Diakinesis of male meiosis of *Skwalacompacta*. Arrows – indicate sex chromosomes bivalent. Scale bar: 5 μm.

At prophase of male meiosis, chromosomes form 13 bivalents (Fig. 4). The twelve bivalents are symmetrical. The large and medium size autosomes form 1–2 chiasmata, and the small bivalents form only one chiasma (Fig. 4).

Sex chromosomes are usually joined by the terminal regions of the long arms (Fig. 4a), although in some cases, the connection between them is not visible (Fig. 4b). During the prophase of meiosis, the X- and Y-chromosomes are always located next to each other suggesting conjugation between them is conserved.

**Figure 5.**
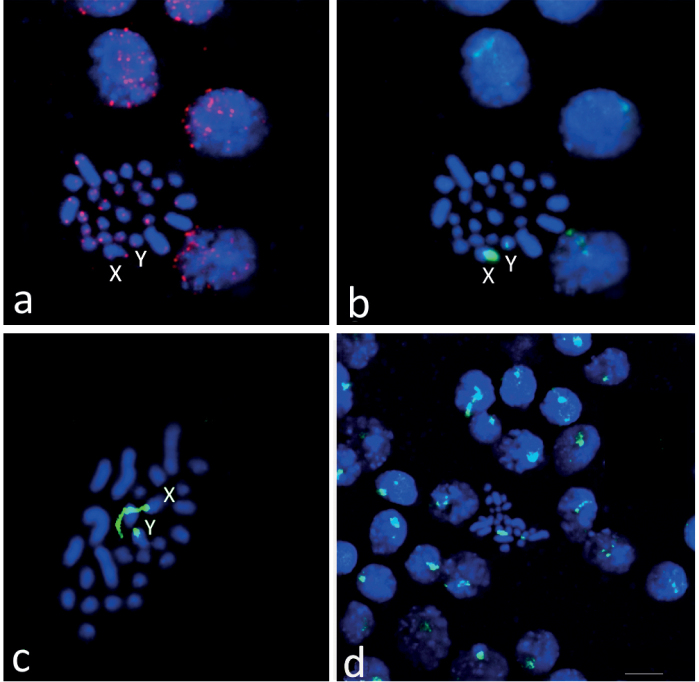
FISH with the telomeric (TTAGG)_n_ probe (red signals) and the ribosomal DNA probe (green signals) on the chromosomes of male *Skwalacompacta*. Same spermatogonial metaphase (**a, b**), early spermatogonial metaphase (**c**) and cells in the interphase and spermatogonial metaphases stages (**d**). Chromosomes were counterstained with DAPI (blue). Scale bar: 5 μm.

Telomeric DNA (TTAGG)_n_ repeats were detected in the terminal regions of all chromosomes (Fig. 5a).

18S rDNA gene clusters were detected only on X and Y chromosomes (Fig. 5b, c). In the X-chromosome, the rDNA cluster is large, occupying the entire short arm and the proximal part of the long arm. This is clearly visible in the early stages of spermatogonial metaphases (Fig. 5c). In the Y chromosome, the rDNA cluster occupies the entire short arm and the proximal part of the long arm (Fig. 5b, c). The rDNA clusters on interphase cells are clearly visible (Fig. 5d).

## ﻿Discussion

To date, karyotypes of only 16 species of Plecoptera belonging to the families Perlidae and Perlodidae have been described (Table 1). The number of chromosomes in karyotypes of Plecoptera species varies from 2n♂=10 in *Perlaimmarginata* (Nakahara 1919) to 2n♂=33 in *Perlodesintricatus* (Matthey and Aubert 1947). In most cases karyotypes were examined in males only. The seven of the fifteen previously studied male stoneflies have 26 chromosomes, and sex is defined as ♂X_1_X_2_0. In females, 14 chromosomes are sometimes indicated in the haploid set. Only in *Paragnetinaimmarginata* the mechanism of XY sex determination is described (Table 1).

**Table 1.** Karyotype features of the Plecoptera species^1^.

Species	2n	n	Sex chromosomes	References
**Perlidae**				
*Acroneuriajezoensis* Okamoto (*Calineuriajezoensis* (Okamoto, 1912))	25♂ 26♀	12, 13♂	X0♂	Itoh 1933
*Perlaabdominalis* Guérin-Méneville, 1838	26♂	–	X_1_X_2_0♂	Matthey and Aubert 1947
*Perlacephalotes* Curtis, 1827 (*Perlabaetica* Rambur, 1842 *Dinocrascephalotes* (Curtis, 1827))	26♂	12, 14♂	X_1_X_2_0♂	Matthey and Aubert 1947
*Perlabipunctata* Pictet, 1833	21♂	11, 10♂	X0♂	Matthey and Aubert 1947
*Paragnetinaimmarginata* (Say, 1823)	10♂	5♂	XY♂	Nakahara 1919
*Perlamarginata* (Panzer, 1799)	22♂ 24♀	10, 12♂	X_1_X_2_0♂	Junker 1923
*Perlamaxima* (Scopoli, 1763) (*Perlamarginata* (Panzer, 1799))	19♂	9, 10♂	X0♂	Matthey and Aubert 1947
**Perlodidae**				
*Isoperlagrammatica* (Poda, 1761)	26	12, 14♂	X_1_X_2_0♂	Matthey and Aubert 1947
*Isoperlarivulorum* (Pictet, 1841)	26♂		X_1_X_2_0♂	Matthey and Aubert 1947
Isogenus (Dictyogenus) imhoffi Pict.	26♂	14	X_1_X_2_0♂	Matthey 1946
Isogenus (Dictyogenus) alpinum (Pictet, 1841) *(Dictyogenusalpinum* (Pictet, 1841))	26♂	14♂	X_1_X_2_0♂	Matthey 1946
Isogenus (Dictyogenus) fontium (Ris) (*Dictyogenusfontium* (Ris, 1896))	26♂	13♂	X_1_X_2_0♂	Matthey and Aubert 1947
*Perlodesintricata* (Pictet, 1841)	33♂	–	–	Matthey and Aubert 1947
*Perlodesjurassicus* Aubert, 1946	31♂	17♂	X_1_X_2_X_3_♂	Matthey 1946
*Perlodesmicrocephalus* (Pictet, 1833)	27♂	15♂	X_1_X_2_X_3_♂	Matthey 1946
*Skwalacompacta* McLachlan, 1872	26♂	13♂	XY♂/XX♀	This paper

^1^ The current valid names of Plecoptera species are given in parentheses according to the Plecoptera Species File. https://plecoptera.speciesfile.org.

*S.compacta* studied by us belongs to the group of species with 2n = 26 and an XX/XY (female/male) mechanism for sex determination. The analysis of the mechanisms of sex chromosome determination in stoneflies shows that in most cases only males were studied, and the mechanism in females was reconstructed from sex chromosomes of males.

For 10 out of the 16 species studied, a ♂X_1_X_2_0 mechanism for sex determination is given, whereas only *Acroneuriajezoensis* (Itoh 1933) and *Perlamarginata* (Junker 1923) have a reliably described female karyotype. Our data on *S.compacta* show the importance of studying both males and females to correctly determine the sex chromosome mechanism in a particular species. Based on the presence of two heterosomes in males of *S.compacta*, we could interpret their sex chromosome mechanism as X_1_X_2_0 (2n=26), and, thus, the female mechanism as X_1_X_1_X_2_X_2_ (2n=28) and only the analysis of the female karyotype (2n=26) allowed us to reliably determine the mechanism in this species as XX/XY.

Other variants of chromosomal sex determination identified in stoneflies based on the analysis of male meiosis alone are as the following: ♂X0 (three species); ♂X_1_X_2_0 (ten species); ♂X_1_X_2_X_3_ (two species) and ♂XY (one species) (Table 1). Therefore, all these data need to be verified with the obligatory study of the karyotypes of females.

The evolution of chromosomal sex determination is probably the most intriguing problem in comparative cytogenetics of the Plecoptera. Analyzing the primary data on karyotypes of stoneflies, the famous cytogeneticist M. J. White emphasized: “A most interesting series of sex chromosome mechanisms exist in the Stone-flies (Plecoptera), but its evolutionary history can hardly be guessed at, on the basis of the available evidence” (White 1973, p. 674).

However, he also emphasized that the behavior of the sex chromosomes in this group during the first meiotic division appears to be very peculiar, whether or not there is a ‘multiple’ mechanism: “Certain species of Stone-flies such as *Perlamaxima*, *P.bipunctata* and *Acroneuriajezoensis* are simply X0 in the males (Aubert and Matthey 1943; Matthey 1946; Matthey and Aubert 1947; Itoh 1933), the X is a large metacentric element which is negatively heteropycnotic and lies in one-half of the first meiotic spindle” (White 1973, p. 674–675).

Indeed, compared to other groups of Polyneoptera, in which sex chromosomes in meiosis are either positively heteropyknotic (Acridoidea) or do not differ in compaction from autosomes (Tettigonioidea) (White 1940), in *S.compacta* we studied, the sex chromosomes are also negatively heteropyknotic during meiotic prophase.

Since White’s time, the peculiarities of chromosomal sex determination in the stoneflies have been discussed numerous times (White 1941; White 1973; Blackman 1995; Blackmon et al. 2017), but always in speculative tones because, new data simply have not been forthcoming since 1947 (Matthey and Aubert 1947).

Without new comparative material, we cannot yet discuss the ways in which sex determination mechanisms are formed. Therefore, we decided to focus on obtaining new information on the karyotypes of stoneflies, using methods that have not been previously applied to the study karyotypes of this group of insects.

At this stage, to study the karyotypic features of one of the most common species of stoneflies in Siberia, *S.compacta*, we tested various methods of preparing chromosome slides from different tissues of larvae and adults (testes and ovaries, Malpighian tubules, pyloric glands of the stomach and neuroblasts of the brain). The method of preparing slides from cell suspension prepared from germarium of testes and ovaries of this species proved to be the most effective (see section Methods).

This approach is a modification of the technique for obtaining chromosome preparations from grasshopper embryos (Bugrov et al. 2001). The technique used makes it possible to achieve a satisfactory spread of cells on glass, which allows to use different methods of chromosome staining depending on the task of the study.

Using this method it was possible to obtain information on the number and morphology of chromosomes of the model species, and, for the first time for the order Plecoptera as a whole, to identify the localization of constitutive heterochromatin (C-blocks) in chromosomes (see section Results).

The use of the C-banding staining method allowed us not only to reveal the relative size and localization of C-heterochromatin in the chromosomes of the studied species, but also to show that one of the arms of the X chromosome is completely heterochromatic, the length of which strongly depends on the degree of spiralization during spermatogonial mitosis (Fig. 3). It should be especially emphasized that the other chromosomes do not exhibit this feature during mitosis.

Fluorescence in situ hybridization (FISH) with telomeric (TTAGG)_n_ sequences revealed strong hybridization signals colocalized with the ends of metaphase chromosomes (Fig. 5a). Our data are in full agreement with the findings of a study of telomeric repeats in the stonefly *Perlaburmeisteriana* Claassen, 1936 (Frydrychová et al. 2004). We can only regret that the authors of this study could not obtain information on other karyotypic features of the studied species and limited themselves to the observation that spermatogonia of this species are “with large numbers of chromosomes” (Frydrychová et al. 2004, p. 173).

This type of localization of telomeric repeats is typical for insect chromosomes (Frydrychová et al. 2004; Kuznetsova et al. 2019). Only in some cases, telomeric repeats appear in an interstitial position, indicating possible inversions and translocations of chromosomes in the karyotypic evolution of a particular group of insects (Jetybayev et al. 2012; Kuznetsova et al. 2019).

The localization of rDNA on stonefly chromosomes has not been previously studied. We identified clusters of rDNA only in the heterochromatic arms of the X and Y chromosomes. It is likely that these arms, rich in rDNA sequences, and are regions of the nucleolus organizer (NOR).

This is also evidenced by strong variations in the relative sizes of these heterochromatic arms at different stages of the cell cycle (Fig. 3). Thus, at the early stage of spermatogonial metaphase, heteromorphism in a pair of sex chromosomes is clearly manifested due to a different degree of amplification of rDNA. Such heteromorphism, for example in amphibians, is often considered as a feature that allows such chromosomes to be considered sex chromosomes (Mahony 1991).

In conclusion, the mechanism of sex determination in stoneflies is the most intriguing problem in the cytogenetics of this group of insects.

As our study has shown, this problem can be alleviated by the use of modern chromosomal analysis techniques.
